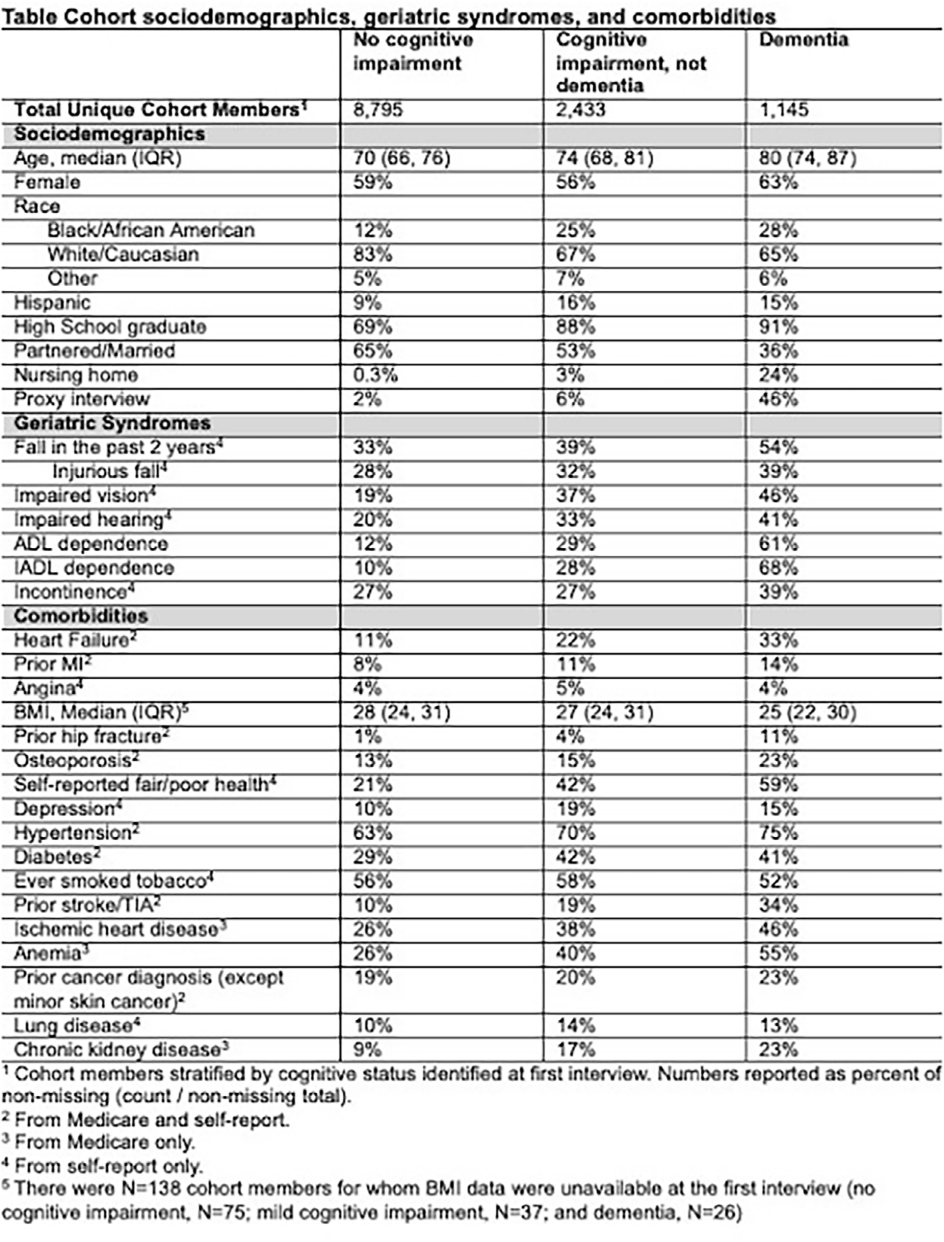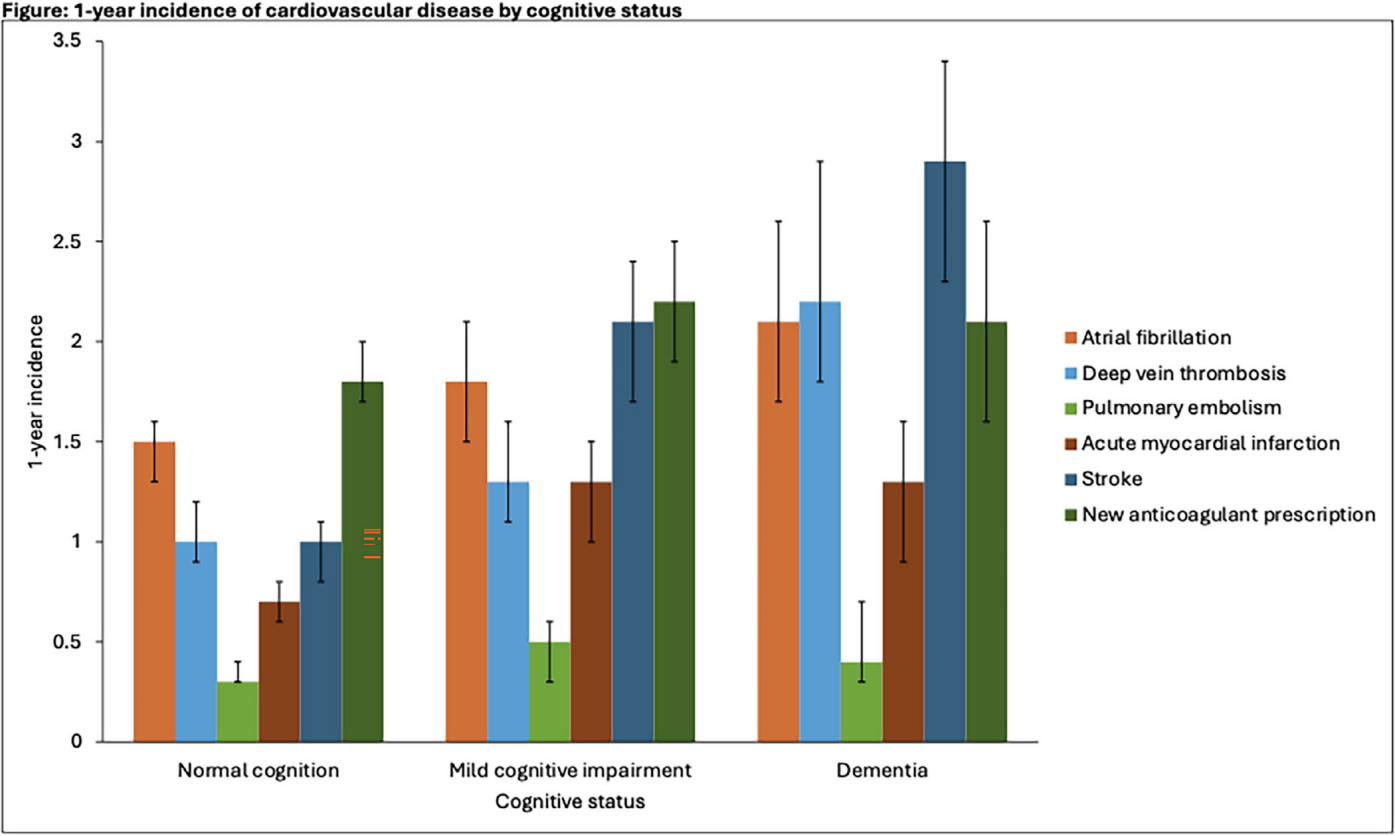# Rates of new indication for antithrombotic drugs in people with cognitive impairment: implications for anti‐amyloid monoclonal antibody treatment

**DOI:** 10.1002/alz70860_103167

**Published:** 2025-12-23

**Authors:** Anna L Parks, Jacquelyn M Lykken, Meghan L Rieu‐Werden, Darae Ko, Dae Kim, Margaret C Fang, Steven M. Greenberg, Daniel M Witt, Mark A. Supiano, Sachin J Shah

**Affiliations:** ^1^ University of Utah, Salt Lake City, UT, USA; ^2^ Massachusetts General Hospital, Boston, MA, USA; ^3^ Hebrew SeniorLife Marcus Institute for Aging Research, Boston, MA, USA; ^4^ Boston Medical Center, Boston, MA, USA; ^5^ University of California, San Francisco, San Francisco, CA, USA

## Abstract

**Background:**

Anti‐amyloid monoclonal antibody treatments may interact with anticoagulants and thrombolytics to increase the risk of intracranial hemorrhage (ICH). Expert guidance advises against co‐prescribing antithrombotic drugs due to increased ICH risk. However, because new cardiovascular diagnoses are common in older adults, some people may develop an indication for anticoagulant or thrombolytic therapy during an anti‐amyloid treatment course. Our goal was to estimate how many people with mild cognitive impairment (MCI) or dementia develop a new condition for which antithrombotic therapy is indicated.

**Method:**

We conducted a longitudinal cohort study using the nationally representative Health and Retirement Study (HRS) data (2010‐2020). We included adults aged ≥65 years who consented to Medicare claims linkage and had no indication for anticoagulants in the 12 months prior to a baseline interview. Participants’ cognition was categorized as normal, MCI (defined in HRS as mild or moderate cognitive impairment without dementia), or dementia. We fit Fine‐Gray survival models accounting for competing risk of death to estimate the 1‐year incidence of atrial fibrillation (AF), deep vein thrombosis (DVT) or pulmonary embolism (PE), acute myocardial infarction (MI), stroke, and new anticoagulant prescriptions. Separate models were fit for each outcome, and models used a robust sandwich estimator to account for repeated observations.

**Result:**

The study included 22,373 participants (Table; median age 71 years, 59% female). Among participants with MCI, the 1‐year incidence was 1.7% for AF, 1.2% for DVT, 0.4% for PE, 1.2% for AMI, 2.0% for stroke, and 2.2% for new anticoagulant prescription (Figure). Overall, the 1‐year likelihood of developing a new indication for anticoagulants or thrombolytics for any reason was 6.9%. Among participants with dementia, 1‐year rates were 1.7% for AF, 1.8% for DVT, 0.3% for PE, 1.0% for AMI, 2.4% for stroke, 1.9% for new anticoagulant prescription, and 7.6% for any indication.

**Conclusion:**

In this national sample of people with MCI or dementia, over 1 year, 7‐8% of people were newly diagnosed with a cardiovascular disease requiring antithrombotic therapy. The risk of developing a condition for which anticoagulants or thrombolytics are indicated should be incorporated into discussions about anti‐amyloid treatment to enhance shared decision‐making.